# Somatosensory Gating Responses Are Associated with Prognosis in Patients with Migraine

**DOI:** 10.3390/brainsci11020166

**Published:** 2021-01-28

**Authors:** Fu-Jung Hsiao, Wei-Ta Chen, Yen-Feng Wang, Shih-Pin Chen, Kuan-Lin Lai, Hung-Yu Liu, Li-Ling Hope Pan, Shuu-Jiun Wang

**Affiliations:** 1 Brain Research Center, National Yang-Ming University, Taipei 112, Taiwan; chensp1977@gmail.com (S.-P.C.); hope881212@gmail.com (L.-L.H.P.); sjwang@vghtpe.gov.tw (S.-J.W.); 2 School of Medicine, National Yang-Ming University, Taipei 112, Taiwan; yfwang851106@gmail.com (Y.-F.W.); laikuanlin@gmail.com (K.-L.L.); christie393@yahoo.com.tw (H.-Y.L.); 3 Department of Neurology, Neurological Institute, Taipei Veterans General Hospital, Taipei 112, Taiwan

**Keywords:** somatosensory gating, episodic migraine, chronic migraine, prognosis, magnetoencephalography

## Abstract

Sensory gating, a habituation-related but more basic protective mechanism against brain sensory overload, is altered in patients with migraine and linked to headache severity. This study investigated whether somatosensory (SI) gating responses determined 3-months treatment outcomes in patients with episodic migraine (EM) and chronic migraine (CM). A 306-channel magnetoencephalography (MEG) with paired-pulse stimulation paradigm was used to record their neuromagnetic responses. To calculate the peak amplitude and latency and compute the gating ratios (second vs. first amplitude), the first and second responses to the paired stimuli from the primary somatosensory cortex were obtained. All patients were assigned to subgroups labeled good or poor according to their headache frequency at baseline compared with at the third month of treatment. The gating ratio in the CM group (*n* = 37) was significantly different between those identified as good and poor (*p* = 0.009). In the EM group (*n* = 30), the latency in the second response differed by treatment outcomes (*p* = 0.007). In the receiver operating characteristic analysis, the areas under the curve for the CM and EM groups were 0.737 and 0.761, respectively. Somatosensory gating responses were associated with treatment outcomes in patients with migraine; future studies with large patient samples are warranted.

## 1. Introduction

Migraine place a substantial clinical and economic burden on both individuals and society [1]. Prognosis prediction is paramount in precision medicine and, more specifically, for the preventive treatment of migraine. In patients with migraine, remission refers to complete freedom from symptoms or the reduced occurrence of attacks over a prolonged period; persistence refers to the continuation of attacks without major changes in frequency, severity, or symptom profile; and progression refers to the increase in attack frequency and disability over time. Numerous studies have established that demographic, clinical, psychological, and social factors may affect both prognosis and treatment outcomes in patients with migraine [2]. Recent studies have suggested potential associations between brain structural changes and treatment outcomes in this population [3,4,5]. However, the association between neurophysiological features and migraine prognosis remains unclear.

Central sensitization is associated with migraine neuropathology [6] and is characterized by impaired response habituation to repetitive afferent stimuli [7,8]. In two of our recent studies, primary somatosensory (SI) gating, a habituation-related but more basic protective mechanism against brain sensory overload, was altered in patients with migraine and linked to the severity of their migraine [9,10]. Specifically, the gating ratio in the SI area, which reflects the extent of cortical disinhibition, increased on a continuum across different participant groups: controls, patients with episodic migraine (EM), and patients with chronic migraine (CM). Moreover, patients with migraine were positively correlated with headache frequency. Our findings from those studies led us to postulate that somatosensory gating responses may constitute a brain signature of migraine severity and diagnosis. Notably, cortical excitability and habituation have been linked to the treatment effect in patients with migraine [11,12,13]. Therefore, the potential of baseline gating responses to predict treatment outcomes is worthy of further investigation.

We hypothesized that SI gating responses at baseline would be associated with migraine prognosis, and that the electrophysiological signatures for treatment outcome should differ between the type of migraine (CM or EM) for their different sensory gating profile. To assess source-level cortical activation of sensory gating responses and directly determine neural activity in the SI cortex, we combined magnetoencephalography (MEG) recording with paired-pulse electrical stimulation. In the localization and measurement of brain activity, MEG is superior to conventional scalp electroencephalography [14]. Our objective was to investigate the association of SI gating responses with outcomes in patients with EM and CM after 3 months of preventive treatment.

## 2. Materials and Methods

### 2.1. Participants

Patients with EM and CM, aged 20–60 years, were enrolled from the headache clinic of Taipei Veterans General Hospital, and were diagnosed according to the International Classification of Headache Disorders, Third Edition (beta version) [15]. Patients were excluded for (i) medication overuse for headaches or the use of migraine prophylactics, (ii) the use of hormones or other medications on a regular or daily basis, and (iii) a history of systemic or major neurological and psychiatric diseases. All of the participants’ results on the physical and neurological examinations and the structural brain magnetic resonance imaging (MRI) scans were normal. The hospital’s institutional review board approved the study protocol (VGHTPE: IRB 2015-10-001BC), and each participant provided written informed consent.

### 2.2. Study Design

Each patient completed a semistructured questionnaire on demographic characteristics and headache profiles on their first outpatient visit. Specifically, the questions on headache profile concerned headache severity (number of headache days per month), days with painkiller use per month, and disease duration (months). Moreover, the Migraine Disability Assessment (MIDAS) questionnaire was administered to assess migraine-related disability [16]. Anxiety and depression were evaluated using the Hospital Anxiety and Depression Scale (HADS) [17]. All patients maintained the headache diary after recruitment.

Each participant underwent a scheduled MEG session. For patients with migraine, the recording was conducted during the interictal period, arbitrarily defined as a period without an acute migraine attack within 2 days before (days −1 and −2) and after (days +1 and +2) the session (day 0). Presence of background or interval headaches during this period was allowed for CM patients. The session was rescheduled in cases of an acute migraine attack during this period or the use of analgesics, triptans, or ergotamines for any reason within 48 h before the recording. The temporal relationship between MEG recordings and migraine attacks was determined from the patient’ headache diaries and through follow-up phone calls. Notably, all patients in Taiwan can visit a tertiary medical center via a direct walk-in, as there is no strict referral system, so many patients with CMs are treatment-naïve in the Neurological Institute of Taipei Veterans General Hospital. Moreover, Taiwan used to have the highest incidence and prevalence rates of end-stage renal disease in the world [18]. Many patients refrain from analgesics use for clinical pain conditions in consideration of the renal side effects of analgesics. Thus, we were able to enroll CM patients without medication overuse, and we used the same inclusion criteria as in a series of our earlier studies [9,13,19,20,21,22].

After the recordings, 5 mg of flunarizine was administered to all patients, which was more effective than topiramate for CM prophylaxis in an 8-week study in CM conducted by our group [23]. Moreover, flunarizine was generally well tolerated and had a safety profile comparable to that of topiramate. At 3-months follow-up, the participants were classified into subgroups of good or poor outcomes according to their headache frequency (i.e., headache days per month) at baseline compared with at the third month of treatment. Good and poor outcomes were defined as a reduction of ≥50% and of <50% in the number headache days per month, respectively.

### 2.3. MEG Recordings

A whole-scalp 306-channel neuromagnetometer (VectorviewTM, Elekta Neuromag, Helsinki, Finland) was used to record brain activity. The head position was represented by four coils placed on the participant’s scalp; moreover, the landmarks (i.e., the nasion and the left and right preauricular points) were determined using Cartesian coordinates and a three-dimensional digitizer. Approximately 100 additional scalp points were also digitized to ensure accurate registration. These landmarks allowed the further registration of the MEG and MRI coordinate systems. Two electrodes were attached above and below one eye to detect electrooculographic activity. During the recording, the participants sat comfortably with their head supported against the neuromagnetometer helmet.

During somatosensory evoked field (SEF) recordings, paired electrical stimuli were delivered to the left index finger using an electrical stimulator (Konstant-Strom Stimulator, Digitimer, Welwyn Gar-den City, UK). The stimuli comprised two 0.2-ms constant-current square-wave pulses, with an interstimulus interval (ISI) of 500 ms and an interpair interval of 8 s [24]. The stimulus intensity was set as two times the subjective sensory threshold, at which no pain response or visible twitches of the flexor digitorum superficialis were elicited. During the SEF recordings, participants were asked to keep their eyes closed and avoid focusing on the electrical stimulation. The signal digitization rate was 600 Hz. The length of each recorded epoch, including a prestimulus baseline of 100 ms, was 500 ms. Epochs that were substantially contaminated by electrooculogram signals (>300 μV) or MEG artifacts (>3000 fT/cm) were excluded from further analysis. At least 100 artifact-free SEF responses to the first and second pulses of the paired stimuli (hereafter “first response” and “second response”, respectively) were averaged online.

### 2.4. MEG Data Analysis

The source analysis of the SEF data was performed using weighted minimum norm estimation (MNE), which provides the current density dynamics of distributed cortical sources. The analysis procedure was described previously [24] and is summarized as follows. First, a forward model was constructed from the MRI-derived surface model of each participant’s brain. The forward model describes the signal pattern generated by a unit dipole at each allowed location on the surface. The topographical three-dimensional representation of the brain was segmented using the BrainVISA software platform (BrainVISA 4.0.2, http://brainvisa.info, French). Subsequently, the anatomical MRI and reconstructed cortical surface were coregistered with the corresponding MEG data set. Second, the inverse operator from the MNE analysis was calculated using the following parameters and specifications: A depth weighting algorithm was used to compensate for the bias in the source calculation. The source orientations were normal to the cortical surface, and a regularization parameter (λ^2^ = 0.33) was used to minimize numerical instability and noise interference as well as to obtain a spatially smoothed solution. Noise covariance was derived from the data at the baseline period (from −100 to 0 ms). Brainstorm software [25] was used for these analyses.

In our two recent studies mentioned earlier, SI gating responses in patients with CM and EM were characterized by alterations in the contralateral SI area [9,10]. Therefore, the contralateral SI cortex was selected as the region of interest in the present study. For both the first and second responses, the peak amplitudes and latencies of cortical sources were extracted from the SI cortex. The ratio of the amplitude in the second response to that in the first response (i.e., second response amplitude/first response amplitude) was used as the gating ratio.

### 2.5. Statistical Analysis

The demographics and clinical characteristics of the participants were assessed using a chi-squared test or analysis of variance (ANOVA) for the factors of group (CM vs. EM) and 3-months treatment outcomes (good vs. poor). ANOVA was also used to compare the gating ratios and the amplitude and latency in the first and second responses. Logistic regression model adjusting for age, sex, anxiety and depression was examined to confirm the significance of prediction. Moreover, receiver operating characteristic (ROC) analysis was used to examine the discrimination ability of sensory gating for predicting treatment outcomes. Bonferroni correction for multiple comparisons was used as necessary, and a *p* value of <0.05 was considered statistically significant.

## 3. Results

### 3.1. Demographic Characteristics and Clinical Profiles

This study recruited 30 patients with EMs and 37 patients with CMs. The clinical and demographic characteristics of the participants are summarized in Table 1. No between-group differences in age or sex were noted. The anxiety scores on the HADS was comparable across the groups, but the scores for depression were higher in the CM group than in the EM group (*p* < 0.05). Notably, the number of monthly headache days was significantly higher in the CM group than the EM group (*p* < 0.001); however, disease duration, days with painkiller use per month, and MIDAS score did not differ significantly between the groups (all *p* > 0.05).

### 3.2. Cortical Responses to Paired-Pulse Stimuli in Patients with EM and CM

**Figure 1:**
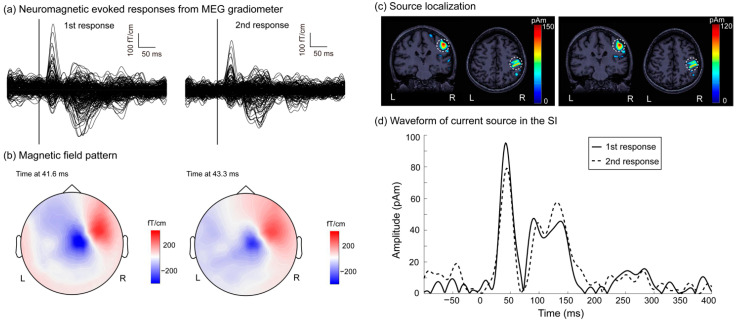
(**a**) Neuromagnetic evoked responses in response to paired stimulation across 204-MEG gradiometer sensors (left, first response; right, second response) in one patient with EMs (EM #1) at −100 to 400 ms. (**b**) Magnetic field patterns of peak latency in the first and second responses at 41.6 and 43.3 ms, respectively. (**c**) Mapping of the source localization of peak latency in the first and second responses onto the individual’s magnetic resonance images. Amplitudes of the cortical sources (color-coded). (**d**) Time-varying amplitude responses in the primary somatosensory area in the first and second responses are shown at −100–400 ms. SI, primary somatosensory cortex.

In response to paired-pulse electrical stimulation, somatosensory evoked magnetic waveforms of all gradiometer channels for the first and second stimulations were superimposed at −100~400 ms, shown in Figure 1a (using a patient with EM #1 as an example). Clear peak responses to stimulation were observed at 40 to 50 ms. Magnetic field patterns in the peak latency exhibited sink-and-source dipolar patterns at 41.6 and 43.3 ms in the first and second responses (Figure 1b). Figure 1c shows the mapping of the peak activation onto the individual’s magnetic resonance images from the MNE source analysis. Cortical sources are clearly observable in the right SI region (in which the amplitude values are color coded). Notably, as presented in Figure 1d, the current density waveform indicates that SI activation in the first and second responses varied over time. The gating effect on the SI responses to the paired-pulse stimulation is also observable.

**Figure 2:**
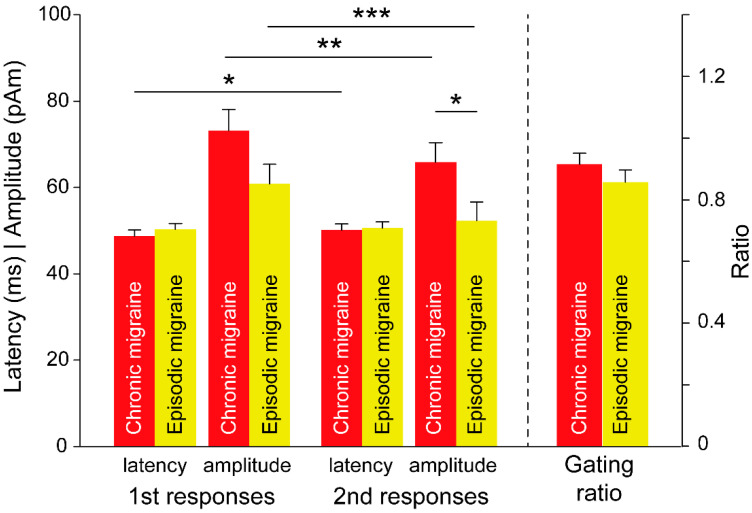
Differences in somatosensory amplitude and latency responses, in the gating ratio between patients with chronic migraine and episodic migraine, and between the first and second responses. *, *p* < 0.05; **, *p* < 0.01; ***, *p* < 0.001.

On the basis of the data on the dynamic cortical activation derived from the MNE analysis, the data on the peak latency and amplitude of SI activation were extracted and compared between the groups as well as between the first and second responses. The latency of the first and second responses and amplitude of the first response were comparable among the patients with CMs and EMs, except for the smaller second amplitude in the EM group (EM = 52.3 ± 4.4 pAm, CM = 65.9 ± 4.5 pAm; *p* = 0.035; Figure 2). Moreover, the gating ratio was higher in the CM group than in the EM group; however, this difference was not significant (EM = 0.858 ± 0.026, CM = 0.916 ± 0.035; *p* > 0.05). In the CM group, a significant difference between the first and second responses was observed; specifically, latency and amplitude were prolonged and reduced in the second response (latency: 48.8 ± 1.3 and 50.1 ± 1.5 ms for the first and second responses, respectively, *p* = 0.024; amplitude: 73.2 ± 4.9 and 65.9 ± 4.5 pAm for the first and second responses, respectively, *p* = 0.004). Amplitude was also reduced in the second response in the EM group (60.9 ± 4.5 and 52.3 ± 4.4 pAm for the first and second responses, respectively, *p* < 0.001).

### 3.3. Between-Subgroup Differences in Treatment Outcomes

According to the 3-months follow-up, 19 and 11 patients in the EM group were arranged into good and poor, respectively. Table 2 summarizes the demographic and clinical characteristics of these patients at baseline. The corresponding characteristics of the EM group were comparable between patients with different outcomes (all *p* > 0.05).

**Figure 3:**
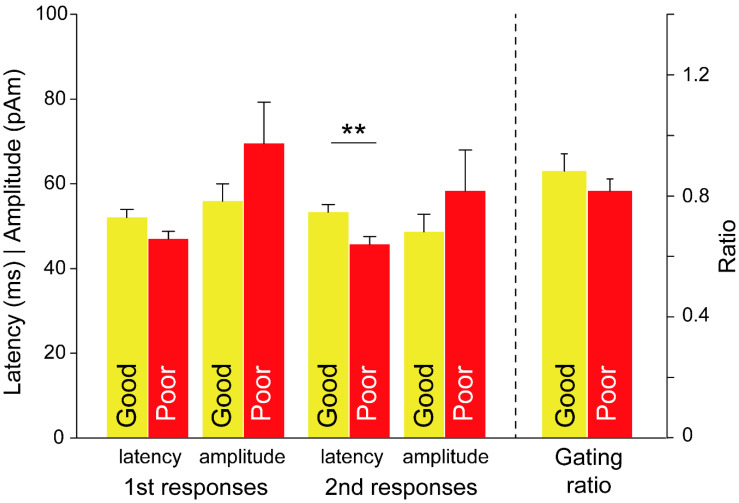
Differences in somatosensory amplitude and latency responses, and in gating ratio in patients with episodic migraine for the effect of treatment outcomes. **, *p* < 0.01.

Differences in the SI gating responses for the factor of treatment outcome were examined with regard to the latency and amplitude in the first and second responses as well as the gating ratio (Figure 3). Among the patients with EMs, latency in the second response was longer in the good subgroup than in the poor subgroup (good: 53.4 ± 1.7 ms, poor: 45.7 ± 1.9 ms; *p* = 0.007). By contrast, between-subgroup differences in latency in the first response were not significant (good: 52.1 ± 1.9 ms, poor: 47.1 ± 1.8 ms; *p* = 0.087). Furthermore, the amplitude and gating ratio did not differ significantly in terms of the outcomes.

In the CM group, 19 and 18 patients were good and poor, respectively. Table 3 summarizes their demographic and clinical characteristics. As with the EM group, these characteristics were comparable between subgroups (all *p* > 0.05).

**Figure 4:**
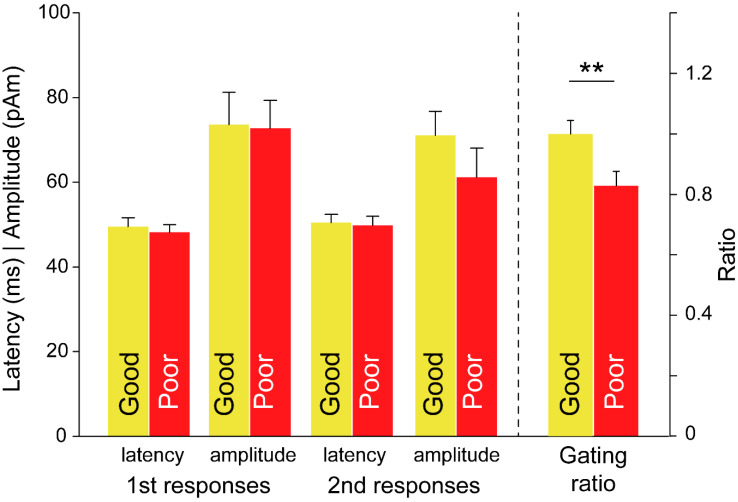
Differences in somatosensory amplitude and latency responses, and in gating ratio in patients with chronic migraine for the effect of treatment outcomes. **, *p* < 0.01.

As with the EM group, the differences in the SI gating responses in the CM group for the treatment outcome factor were examined with regard to the latency and amplitude in the first and second responses as well as the gating ratio (Figure 4). The gating ratio was higher in good than in poor (good: 1.00 ± 0.04, poor: 0.83 ± 0.05; *p* = 0.009). However, no difference for the treatment outcomes was noted in the latency and amplitude responses.

In the logistic regression analysis, the SI gating ratio in CMs was associated with headache outcome after controlling for age, sex, anxiety, and depression (adjusted OR 94.2, *p* = 0.027), as well as the second latency response in EM (adjusted OR 1.2, *p* = 0.024). The ROC analysis revealed that the areas under the curve were 0.737 and 0.761 (95% confidence interval: 0.576–0.898 and 0.583–0.939) for the CM and EM groups, respectively, indicating that the discrimination ability of sensory gating in predicting the treatment outcomes was satisfactory.

## 4. Discussion

SI gating responses in patients with migraine at baseline were linked to the 3-months treatment outcomes despite the homogeneity in age, sex, and baseline psychiatric and clinical (i.e., headache) profiles. Specifically, patients with CMs with less SI inhibition had better outcomes, whereas in patients with EMs, better outcomes were indicated by longer peak latency of SI responses at baseline. Notably, the discriminative ability of SI gating responses in predicting treatment outcomes was satisfactory.

As mentioned, reduced amplitude was observed in the SI gating responses to repetitive stimulation in both patients with CMs and EMs in our recent studies [9,10]. Moreover, the amplitude and latency, as well as the gating ratios, were comparable between the groups, except for the smaller amplitude in the second response in the EM group. Notably, both the mean amplitude and the gating ratio were non-significantly higher in the CM group than the EM group. This is in line with our previous findings that abnormal SI excitability and inhibition in patients with CM are reflective of alternations in sensory modulation and are linked to migraine chronification [9,10]. Furthermore, interneuronal inhibition has been reported in studies on neural substrates of sensory gating to be distributed among the primary sensory cortices, thalamus, prefrontal cortex, hippocampus, and rhinal cortex [24,26,27]. Thus, the atypical SI gating responses in the present study were indicative of abnormalities in cortical–subcortical neural interactions [7] that may fluctuate with migraine status [10,28]. Whether SI gating is an electrophysiological brain signature characterizing clinical phenotypes of migraine remains to be determined [9,10].

In the present study, SI inhibition at baseline determined the outcomes after 3-months preventive medication in the CM group. Specifically, lower SI inhibition was associated with reductions in headache frequency after treatment. Taken together with our previous findings of negative correlations between SI inhibition and headache frequency [9,10], the present findings indicate that deficits of sensory processing (i.e., alterations in cortical excitability and inhibition) in patients with CMs can be attenuated using preventive medication, mitigating the frequency of headache attacks. Notably, the action mechanisms of flunarizine, the prophylactic used in the present study, suppress excitatory nervous signaling using calcium receptors, facilitate the production of chemicals modifying the effect of the neurotransmitter gamma aminobutyric acid, reduce neuronal sensitization, and block cortical spreading depression [29]. In their exploration of the effects of preventive treatment with topiramate on cortical habituation to nociceptive stimulation in patients with migraine, Clemente et al. [11] reported that the treatment modulated cortical excitability and thus the habituation to stimulation. Notably, preventive medication mainly acts on the sensory–discriminative aspect of pain, rather than on the affective dimension (e.g., the anterior cingulate cortex) [11]. Several studies have indicated that migraine may be characterized by atypical brain activation or abnormalities in gray matter volume, particularly in areas mediating affective pain processing, including the salience and limbic networks [30,31,32]. Taken together, the results indicate that SI gating responses at baseline may be associated with the prognoses of patients with migraine.

In patients with EMs, the peak latency of the second response at baseline accounted for differences in treatment outcomes at 3-months follow-up. Specifically, longer peak latency was associated with better treatment outcomes. Notably, although latency in the second response was prolonged in the EM group, it did not differ significantly from that in the CM group, and no significant differences between responses in latency were observed. In a longitudinal study, latency responses to external stimulation slightly increased between blocks in patients with migraine, but did not differ from those in controls [33]. The basis for the prolonged latency is unclear [34]; in that longitudinal study, it was interpreted as a slight but normal habituation of underlying neurophysiological processes [33]. This is supported by our previous finding of comparable inhibitory capacity between patients with EMs and controls [10]. A case study of a patient with migraine revealed that treatment with valproate normalized the prolonged latency of SI responses [35]. This finding is partly the reason that latency changes were later acknowledged as a crucial indicator for migraine classification [36]. This evidence leads us to suggest that SI latency responses to repetitive stimulation in patients with EMs were linked to the 3-months treatment outcomes in this group. The underlying mechanism was the effect of the preventive medication on excitability modulation.

The present study demonstrated that gating responses in the SI cortex may have predicted the patients’ treatment outcomes. Notably, recent studies have independently reported associations between the volume of gray matter in the right hippocampus, orbitofrontal cortex, or cerebellum with headache outcomes in patients with migraine [3,4,5]. The discrepancy between the brain areas identified may be ascribed to differences in fundamentals (functional vs. structural), tasks (evoked vs. resting), and treatment durations (3 months vs. 2 years). Notably, SI function has been separately linked to the treatment effect of topiramate [11] and onabotulintoxinA [12] in patients with migraine. SI activation represents functional alterations and may constitute a potential brain signature for migraine prognosis. Taken together with evidence from neurophysiological research on the pivotal role of sensory pain processing in migraine treatment [37], the impaired habituation to repetitive afferent stimulation that characterizes migraine [7,11,12,36], and the association of the SI cortex with migraine chronification [9,10], the SI cortex appears to be a critical indicator of migraine prognosis.

Some limitations should be addressed in interpreting the present results. First, a systematic review of preventive treatment in migraine reported that sleep, medication overuse, and self-efficacy for managing headaches also constitute potential prognostic factors [2], although the SI gating responses were indicative of significantly different treatment outcomes as the migraine and psychiatric profiles were controlled. Because these prognostic factors (not controlled in the present study) are complex and difficult to handle, further investigations with the use of more deliberate designs are necessary. Second, the SI gating recordings were not performed at 3-months follow-up; in other words, fluctuations in cortical excitability and inhibition after the treatment were not examined. Moreover, the ability of the SI gating responses to differentiate between treatment outcomes must be confirmed using a new sample of patients with migraine.

## 5. Conclusions

SI gating responses are predictive of 3-months treatment outcomes in patients with migraine with good discrimination ability. This finding suggests that migraine preventive treatments may target brain excitability change underpinning migraine pathophysiology. Further studies with larger sample sizes are warranted to confirm the prognostic value of SI gating and its applicability in precision medicine.

## Figures and Tables

**Table 1:** Demographics, psychological characteristics and headache profile in chronic and episodic migraine.

	CM (*n* = 37)	EM (*n* = 30)	*p* Value
Age	36.4 ± 10.7	37.0 ± 10.0	0.83
Sex	34 F/3 M	24 F/6 M	0.28
HADS_A	9.1 ± 4.1	7.4 ± 4.2	0.099
HADS_D	7.3 ± 3.8	5.3 ± 3.1	0.026
Migraine profile			
Headache days (/month)	23.4 ± 6.7	7.5 ± 3.5	<0.001
Duration (month)	229.7 ± 149.0	192.0 ± 125.4	0.289
Days with painkiller (/month)	6.8 ± 8.4	3.4 ± 6.0	0.07
MIDAS	43.1 ± 58.2	32.3 ± 28.2	0.362

CM, chronic migraine; EM, episodic migraine; F, female; M, male; HADS: Hospital Anxiety and Depression Score; A: anxiety; D: depression; MIDAS: Migraine Disability Assessment.

**Table 2:** Demographics, psychological characteristics and headache profile in EMs with different treatment outcomes.

	Good (*n* = 19)	Poor (*n* = 11)	*p* Value
Age	38.5 ± 10.3	34.6 ± 9.6	0.34
Sex	15 F/4 M	9 F/2 M	>0.9
HADS_A	7.7 ± 4.3	6.9 ± 4.2	0.61
HADS_D	5.5 ± 3.1	4.9 ± 3.3	0.61
Migraine profile			
Headache days (/month)	8.4 ± 3.1	6.1 ± 3.9	0.09
Duration (month)	188.0 ± 135.3	199.2 ± 111.9	0.83
Days with painkiller (/month)	4.0 ± 7.2	2.4 ± 3.3	0.49
MIDAS	34.4 ± 29.7	28.7 ± 26.5	0.61

EM, episodic migraine; F, female; M, male; HADS: Hospital Anxiety and Depression Score; A: anxiety; D: depression; MIDAS: Migraine Disability Assessment.

**Table 3:** Demographics, psychological characteristics and headache profiles in CMs with different treatment outcomes.

	Good (*n* = 19)	Poor (*n* = 18)	*p* Value
Age	35.4 ± 12.1	37.4 ± 9.5	0.593
Sex	18 F/1 M	16 F/2 M	>0.9
HADS_A	9.0 ± 2.9	9.3 ± 4.9	0.85
HADS_D	7.1 ± 3.6	7.4 ± 4.1	0.81
Migraine profile			
Headache days (/month)	24.3 ± 7.5	22.7 ± 6.1	0.52
Duration (month)	253.3 ± 169.8	204.7 ± 123.5	0.34
Days with painkiller (/month)	7.4 ± 9.2	6.3 ± 7.7	0.7
MIDAS	50.1 ± 74.7	36.5 ± 37.5	0.49

CM, chronic migraine; F, female; M, male; HADS: Hospital Anxiety and Depression Score; A: anxiety; D: depression; MIDAS: Migraine Disability Assessment.

## Data Availability

The data presented in this study are available on request from the corresponding author. The data are not publicly available due to the confidentiality.
